# Delving into the Perception, Use, and Context of Duloxetine in Clinical Practice: An Analysis Based on the Experience of Healthcare Professionals

**DOI:** 10.3390/brainsci15070757

**Published:** 2025-07-17

**Authors:** Oscar Fraile-Martinez, Cielo Garcia-Montero, Miguel Angel Alvarez-Mon, Miguel A. Ortega, Melchor Alvarez-Mon, Javier Quintero

**Affiliations:** 1 Department of Medicine and Medical Specialities, Faculty of Medicine and Health Sciences, University of Alcalá, 28801 Madrid, Spain; 2 Ramón y Cajal Institute of Sanitary Research (IRYCIS), 28034 Madrid, Spain; 3 CIBERSAM-ISCIII (Biomedical Research Networking Centre in Mental Health), 28029 Madrid, Spain; 4 Department of Psychiatry and Mental Health, University Hospital Infanta Leonor, 28031 Madrid, Spain; 5 Immune System Diseases-Rheumatology and Internal Medicine Service, University Hospital Prince of Asturias, 28805 Madrid, Spain; 6 Networking Research Center on for Liver and Digestive Diseases (CIBEREHD), 28806 Alcala de Henares, Spain; 7 Department of Legal and Psychiatry, Complutense University, 28040 Madrid, Spain

**Keywords:** duloxetine, major depressive disorder (MDD), generalized anxiety disorder (GAD), chronic pain, efficacy, tolerability, adherence, perceived satisfaction

## Abstract

Background and objectives: Duloxetine is widely used for the treatment of major depressive disorder (MDD), generalized anxiety disorder (GAD), and various types of neuropathic pain. While its efficacy is well documented in clinical trials, less is known about how it is perceived and utilized in routine psychiatric practice. To address this knowledge gap, we conducted a cross-sectional observational study involving 80 psychiatrists from Spain to assess real-world clinical attitudes toward duloxetine. Methods: Participants completed a 20-item multiple-choice questionnaire that examined familiarity, perceived efficacy in multiple conditions (MDD, GAD, neuropathic pain, somatization, and quality of life), and perspectives on tolerability, safety, adherence, and overall satisfaction. Results: Survey results indicated that a large majority of psychiatrists frequently prescribe duloxetine, particularly for patients with MDD and comorbid chronic pain. Notably, 94% rated it as either “more effective” or “much more effective” for diabetic peripheral neuropathic pain. Psychiatrists reported a high perceived efficacy of duloxetine: 94% rated it as “more effective” or “much more effective” for diabetic peripheral neuropathy, and 93% gave similarly positive ratings for general neuropathic pain. For somatization, 70% found it “effective” or “very effective”, and 83% observed improvements in quality of life for many of their patients. Psychiatrists generally reported favorable perceptions of duloxetine’s tolerability profile: 97.5% rated it as the antidepressant associated with the least weight gain, and 82.5% perceived fewer sexual side effects compared to other options. Sedation and gastrointestinal side effects were generally considered mild or less severe. In terms of treatment adherence, 69% rated it as “better” or “much better” than other antidepressants, and 80% found its combination with other antidepressants to be “favorable” or “very favorable”. Overall satisfaction was high, with 99% of psychiatrists reporting being either “satisfied” or “very satisfied” with its use. The side effect profile was generally viewed as manageable, with low perceived rates of weight gain, sedation, and sexual dysfunction. Furthermore, 96% of respondents expressed a willingness to recommend duloxetine to their colleagues. Conclusions: Psychiatrists reported highly favorable attitudes toward duloxetine, viewing it as a flexible treatment option in routine care. However, these findings reflect clinicians’ subjective perceptions rather than objective clinical outcomes and should be interpreted accordingly.

## 1. Introduction

Duloxetine is a serotonin and norepinephrine reuptake inhibitor (SNRI) that has become a highly relevant medication for the treatment of various conditions [1]. Among its most important applications, its efficacy in managing psychiatric disorders stands out, particularly in the treatment of major depressive disorder (MDD) [2] and generalized anxiety disorder (GAD) [3]. In addition, duloxetine plays an important role in the treatment of neuropathic pain, especially diabetic peripheral neuropathy [4], widespread pain in fibromyalgia [5], osteoarthritis [6], and other chronic musculoskeletal pain syndromes.

Despite its proven efficacy in controlled clinical trials, there remains a lack of comprehensive data on how duloxetine is perceived and prescribed in everyday clinical practice. Many studies have focused on efficacy and safety under ideal conditions, but fewer have explored the practical challenges clinicians face when integrating duloxetine into complex, real-world treatment regimens, especially considering patient adherence, tolerability, and individualized therapeutic needs [7,8]. In this context, clinicians’ knowledge and experience are essential in determining when and how to use duloxetine, as these factors directly influence therapeutic decisions and personalized treatment planning.

The current therapeutic landscape for MDD, GAD, and chronic pain includes multiple options, requiring clinicians to make ongoing comparisons between medications. Selecting the most appropriate treatment involves a detailed analysis of each drug’s profile, considering factors such as onset of action, duration of therapeutic effect, and the incidence of long-term relapse [9,10]. In addition to assessing a drug’s efficacy, healthcare professionals must also weigh its tolerability and potential adverse effects, which can influence treatment adherence. Symptoms such as nausea, fatigue, weight changes, sexual dysfunction, or cardiovascular effects may lead patients to discontinue medication prematurely, reducing its effectiveness [11]. In this regard, studies support the benefits and safety of duloxetine at doses ranging from 60 to 120 mg per day, depending on the condition, although its side effect profile must be carefully evaluated on a case-by-case basis [1,12].

Another key aspect is the ease of managing treatment, including dosing, interactions with other medications, and the need for adjustments in specific populations such as older adults or patients with medical comorbidities. In this sense, the possibility of combining duloxetine with other therapeutic agents—either within the antidepressant spectrum or in combination with analgesics for chronic pain—enhances its utility but also raises questions about its safety and synergy with other drugs [13,14]. Furthermore, its effectiveness in specific subgroups, such as patients with treatment-resistant depressive symptoms, individuals with severe chronic pain, or those with a history of adverse effects from other antidepressants, is a decisive factor in clinical decision-making. General perceptions of its safety, based on clinicians’ accumulated experience and clinical studies, play a fundamental role in its everyday use and guide the selection of duloxetine in specific therapeutic contexts.

Given these considerations, there is a clear need to fill the existing knowledge gap concerning real-world clinical use of duloxetine. This study addresses this gap by employing a structured 20-item questionnaire designed to assess healthcare professionals’ familiarity with duloxetine, prescribing patterns across different indications, and perceptions of its efficacy and tolerability relative to other antidepressants. We also explore clinicians’ approaches to treatment adherence, adverse effect management, and combination therapies.

Thus, the primary aim of this study is to provide a comprehensive overview of duloxetine’s clinical utilization and acceptance, thereby contributing novel insights that may inform future prescribing practices and guide optimized patient care.

## 2. Materials and Methods

### 2.1. Study Design

This study collected data from an exploratory, cross-sectional, online self-completed survey aimed at psychiatrists working in Spain. The target population consisted of practicing psychiatrists from various hospitals and mental health centers across the country.

Participants were recruited through a non-probabilistic, convenience sampling strategy, using an open call distributed by Adamed Laboratories (Madrid, Spain). The inclusion criteria for participation were (1) being a licensed psychiatrist, (2) having more than five years of clinical experience, and (3) currently practicing psychiatry in Spain. A total of 80 psychiatrists completed the questionnaire. The response rate could not be precisely calculated due to the open recruitment approach and lack of denominator data for the total number of professionals reached.

To collect the information, a structured questionnaire consisting of 20 multiple-choice questions (available in the Supplementary Materials) was developed, covering the following areas: (1) Familiarity with and routine use of duloxetine in the context of major depressive disorder (MDD). (2) Perceived efficacy of duloxetine in various clinical scenarios, including different forms of neuropathic pain, generalized anxiety disorder (GAD), somatization, and impact on quality of life. (3) Safety and tolerability of duloxetine assessed both generally and in relation to specific symptoms. (4) Overall perception of duloxetine: adherence, combination use, and treatment satisfaction

The questionnaire was reviewed by a panel of experts to ensure clarity of wording and content validity. All responses were processed anonymously to guarantee participant confidentiality. Additionally, the response order was randomized during the analysis to minimize bias and preserve the reliability of the results.

### 2.2. Data Analysis

The data obtained were analyzed using descriptive statistics, calculating the frequency and percentage of responses for each questionnaire item. Responses from different phases were compared to identify potential differences in psychiatrists’ perceptions, and recurring patterns were examined. Python code (using the Matplotlib 3.10.3 library) was also employed to generate charts that facilitated the visualization of the results. Given the descriptive and exploratory nature of the study, and the lack of subgroup identifiers among respondents, no inferential statistical analyses were performed.

## 3. Results

### 3.1. Familiarity and Routine Use of Duloxetine in the Context of Major Depressive Disorder

**Figure 1:**
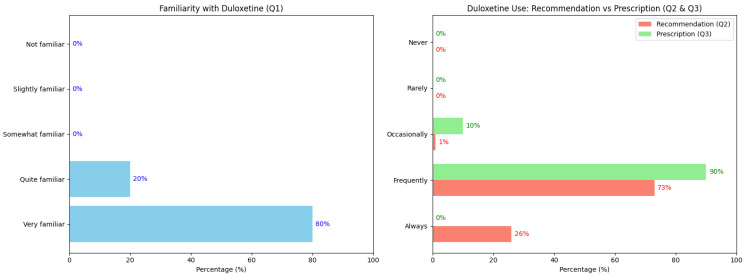
Graphical representation of the results regarding familiarity and use of duloxetine in the context of major depressive disorder (MDD). The figure on the left shows familiarity with the use of duloxetine, the right figure shows recommendations for the treatment of major depressive disorder with chronic pain and prescriptions for MDD.

In a first group of questions, we aimed to assess psychiatrists’ familiarity with and use of duloxetine (questions 1, 2, and 3). Regarding familiarity (question 1), results showed that the vast majority of respondents feel highly comfortable managing duloxetine. A total of 80% reported being “very familiar” and 20% “fairly familiar”, indicating a high level of integration of this drug in clinical practice. The absence of responses indicating low familiarity suggests that professionals possess solid knowledge and experience in handling this medication. Regarding the recommendation of duloxetine for patients with comorbid MDD and chronic pain (question 2), data revealed a strong positive trend: 73% reported recommending it “frequently”, and 26% said “always”, while only 1% reported occasional use. Concerning its use for treating MDD alone (question 3), prescription was very consistent, with 90% prescribing it “frequently”, 10% “occasionally”, and no responses for “rarely” or “never”. The results are summarized in Figure 1. 

### 3.2. Perceived Efficacy of Duloxetine in Various Clinical Contexts

**Figure 2:**
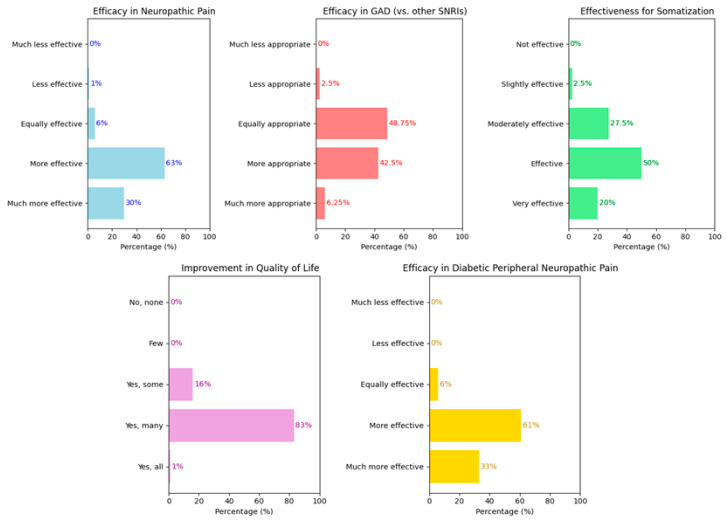
Graphical representation of the results concerning the perceived efficacy of duloxetine in different clinical contexts. (**Top left**): efficacy in neuropathic pain; (**top center**): efficacy in generalized anxiety disorder compared to other SNRIs; (**top right**): efficacy in somatization; (**bottom left**): improvement in quality of life; (**bottom right**): efficacy in peripheral neuropathic pain.

A second cluster of questions explored the perceived efficacy of duloxetine beyond MDD. Specifically, efficacy was assessed in neuropathic pain (question 5), GAD (question 12), somatization (question 13), improvement in quality of life (question 17), and in diabetic peripheral neuropathy (DPN). Results are graphically summarized in Figure 2. For neuropathic pain, most respondents viewed duloxetine as superior, with 63% rating it as “more effective” and 30% as “much more effective” than other antidepressants. Only 6% considered it similarly effective, and just 1% rated it as less effective; no extreme negative responses were recorded. Regarding GAD treatment, responses were more varied: 48.75% found it “equally suitable” and 42.5% “more suitable” than other SNRIs. Only 6.25% rated it “much more suitable” and 2.5% “less suitable”; no respondents chose “much less suitable”. For somatization, 20% rated duloxetine as “very effective”, 50% as “effective”, 27.5% as “moderately effective”, and only 2.5% as “slightly effective”; no respondents rated it as “ineffective”. Regarding quality of life improvements, 83% indicated that “many” of their patients experienced improvement, 16% noted improvement in “some”, and 1% in “all”; no negative responses were reported. For diabetic peripheral neuropathy, perceptions were again favorable: 61% rated duloxetine as “more effective” and 33% as “much more effective” compared to other SNRIs. Only 6% found it “equally effective”, and no negative ratings were recorded.

### 3.3. Safety and Tolerability of Duloxetine

**Figure 3:**
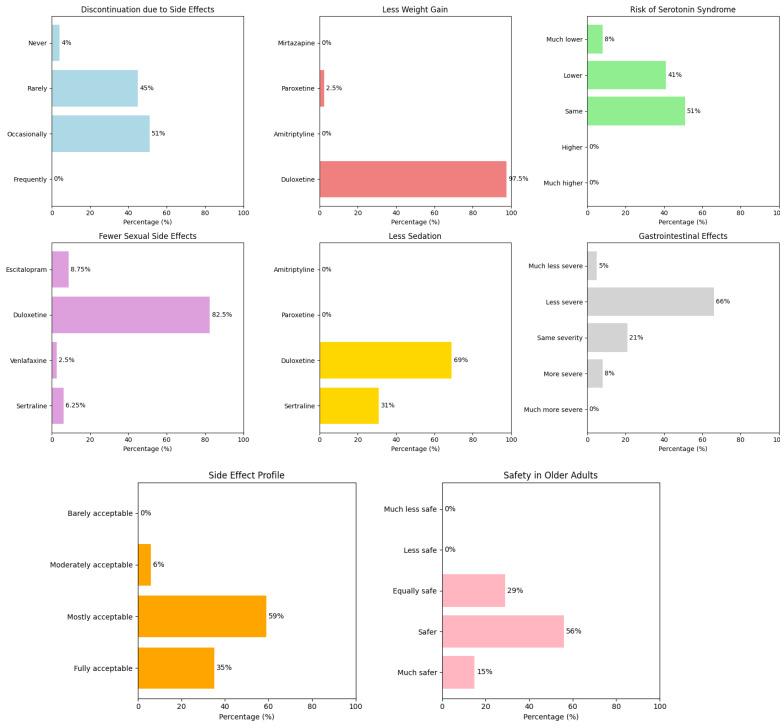
Graphical representation of the results related to the safety and tolerability of duloxetine. In the top row, from left to right: treatment discontinuation due to side effects, weight gain, and risk of serotonin syndrome. In the middle row: sexual side effects, sedation, and gastrointestinal side effects (from left to right, respectively). In the bottom row: overall side effect profile (left) and safety in older adults (right).

A key focus of the survey was the safety and tolerability of duloxetine, including treatment discontinuation due to side effects (question 6), weight gain (question 7), risk of serotonin syndrome (question 8), sexual side effects (question 9), sedation (question 11); gastrointestinal effects (question 15), overall side effect profile (question 19), and safety in older adults (question 20). Figure 3 summarizes the results. For treatment discontinuation due to side effects, 51% had done so occasionally, 45% rarely, and 4% never. No one reported frequent discontinuation. Regarding weight gain, 97.5% of respondents selected duloxetine as causing the least weight gain, compared to 2.5% for paroxetine; amitriptyline and mirtazapine received 0%. On serotonin syndrome risk, 51% felt duloxetine posed equal risk as other SNRIs, 41% considered it lower, and 8% much lower; no one viewed the risk as higher. Regarding sexual side effects, 82.5% found duloxetine caused fewer, followed by escitalopram (8.75%), sertraline (6.25%), and venlafaxine (2.5%). For sedation, 69% selected duloxetine as less sedating, and 31% chose sertraline; none chose paroxetine or amitriptyline. On gastrointestinal side effects, 66% reported these were less severe with duloxetine, 21% equally severe, 8% more severe, and 5% much less severe. No one selected “much more severe”. The overall side effect profile was rated “mostly acceptable” by 59%, “fully acceptable” by 35%, and “moderately acceptable” by 6%; none found it “barely acceptable”. Regarding safety in patients aged 50–75, 56% considered duloxetine “safer” than other SNRIs, 15% “much safer”, and 29% “equally safe”. No one considered it “less safe” or “much less safe”.

### 3.4. General Perception of Duloxetine: Adherence, Combination Use, and Treatment Satisfaction

**Figure 4:**
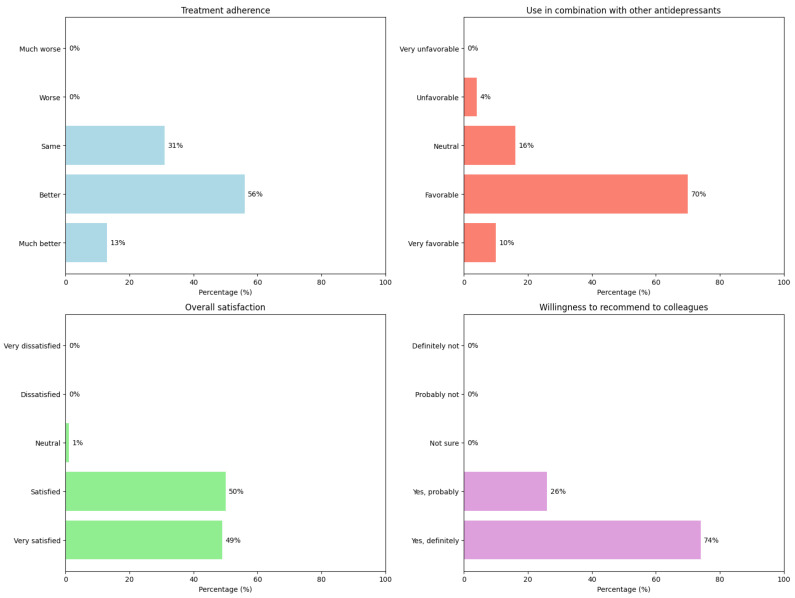
Graphical representation of treatment adherence and overall perception of duloxetine. (**Top left**): treatment adherence; (**top right**): use in combination with other antidepressants; (**bottom left**): overall satisfaction; (**bottom right**): recommendation to colleagues.

Finally, we explored adherence (question 4) and overall perception, including combination with other antidepressants (question 10) and general satisfaction with duloxetine (question 14). Respondents were also asked whether they would recommend duloxetine to colleagues (question 16). For adherence, 13% rated duloxetine adherence as “much better”, 56% “better”, and 31% “equal” to other antidepressants. None rated it “worse” or “much worse”. On combining duloxetine with other antidepressants, 10% rated this as “very favorable”, 70% “favorable”, 16% “neutral”, and 4% “unfavorable”; none rated it “very unfavorable”. Regarding overall satisfaction, 49% were “very satisfied”, 50% “satisfied”, and 1% “neutral”; no respondents expressed dissatisfaction. On recommending duloxetine to colleagues, 74% answered “yes, definitely”, and 26% “yes, probably”, with no negative or uncertain responses. In Figure 4, commented results are summarized.

## 4. Discussion

In this study, we explored key issues related to the use and perception of duloxetine among medical professionals specializing in psychiatry. Overall, the survey results reflect psychiatrists’ perceptions that are broadly in line with previously published findings, though they do not represent objective clinical data. Below, we discuss some of the most relevant findings derived from our results.

The high degree of familiarity and routine use of duloxetine among participating psychiatrists—with 80% reporting being “very familiar” and 20% “fairly familiar”—demonstrates that this medication is well integrated into clinical practice for the treatment of MDD and other conditions. Specifically, 96% of the psychiatrists reported prescribing duloxetine always or frequently for MDD patients with chronic pain. This is consistent with scientific literature indicating duloxetine’s efficacy in patients presenting both affective and somatic symptoms [15,16]. According to real world data, duloxetine could be more effective when compared to other antidepressants such as selective serotonin reuptake inhibitors (SSRIs) in certain subgroups, including elder individuals (>60 years), patients suffering from a first depressive episode, those with higher baseline painful symptoms score levels, and those with more severe baseline depression [17,18].

However, therapeutic success depends not only on pharmacological efficacy but also on the broader clinical context. For example, one study showed that collaborative care, combined with either placebo or duloxetine, produced better outcomes in patients with MDD and (sub)chronic pain compared to duloxetine alone [19]. Duloxetine should therefore be considered as part of a comprehensive treatment framework.

Additionally, 90% of respondents reported prescribing duloxetine “frequently” for MDD, reflecting strong confidence in this drug and positioning it as a key tool in the management of depression. Evidence supports the efficacy and tolerability of duloxetine at a dose of 60 mg QD during both acute and relapse-prevention phases of MDD [12]. Although some clinicians use higher doses (90–120 mg/day) in severe or resistant cases, studies suggest limited added benefit at higher doses and an increased risk of side effects [12].

Beyond MDD, duloxetine was also perceived as effective in treating neuropathic pain. Most respondents rated it as more effective than other antidepressants, a view supported by the literature, which identifies duloxetine (60–120 mg/day) as a first-line option for neuropathic pain [20]. A systematic review and meta-analysis by Birkinshaw et al. [10] found that out of 25 antidepressants assessed across 176 studies with 28,664 patients, duloxetine was the only one with consistent evidence for efficacy in chronic pain conditions such as neuropathic pain, fibromyalgia, and musculoskeletal pain.

Similarly, duloxetine was widely rated as one of the most effective options for diabetic peripheral neuropathy. This finding is supported by systematic reviews and meta-analyses that recommend duloxetine as a first-line treatment for this indication [21,22].

In contrast, perceptions of its use for generalized anxiety disorder (GAD) were more mixed. While 42.5% rated it as more appropriate than other SNRIs, nearly half considered it equally appropriate. These discrepancies may reflect individual variation in response and tolerability, which is well-documented in the literature [23,24]. Although duloxetine is approved for GAD, comparative studies with other SNRIs such as venlafaxine or SSRIs like escitalopram show mixed results, which may explain divergent clinical opinions [25]. Also, a systematic review and network meta-analysis of double-blind randomized controlled trials conducted by Kong et al. [26] reported that duloxetine was associated with better remission in GAD patients compared to placebo and other therapeutic alternatives; however, it was worse than placebo in terms of tolerability, further supporting the findings obtained in our study.

Regarding somatization, 70% of respondents considered duloxetine effective or very effective, suggesting that its dual action on serotonin and norepinephrine may benefit patients with somatic symptoms in MDD or GAD [27,28].

Another key finding was the perceived improvement in patients’ quality of life. Most psychiatrists observed meaningful gains in functioning and well-being, beyond symptom relief [29,30,31]. This reflects a holistic clinical benefit, consistent with previous findings on duloxetine’s impact on physical and emotional health domains.

Respondents reported a favorable safety and tolerability profile. Most stated they “occasionally” or “rarely” discontinued duloxetine due to side effects, and none reported “frequent” discontinuation. These perceptions are in line with data suggesting that side effects associated with duloxetine are generally manageable and seldom lead to treatment dropout [32,33].

Notably, 97.5% of respondents identified duloxetine as the least antidepressant associated with weight gain, in contrast to medications such as paroxetine, amitriptyline, and mirtazapine. This aligns with prior research indicating a more favorable metabolic profile for duloxetine [34] and real-world evidence suggesting that duloxetine’s weight-neutral profile distinguishes it from other antidepressants that commonly cause weight gain [35].

As for serotonin syndrome, most participants rated duloxetine’s risk as equal to or lower than that of other SNRIs. Although duloxetine has been linked to serotonin syndrome in rare cases [36,37], venlafaxine appears to carry a higher risk [38]. Caution is still warranted, particularly in combination with other serotonergic agents.

Regarding sexual side effects, 82.5% of psychiatrists believed duloxetine caused fewer problems than other antidepressants. This is consistent with studies showing duloxetine has a lower incidence of sexual dysfunction than paroxetine [39] and escitalopram [40], which is critical since sexual side effects are a leading cause of non-adherence. This more favorable profile of duloxetine on sexual function was also highlighted by psychiatrists in previous real-world studies [35].

Duloxetine was also rated as less sedating than most other antidepressants (except sertraline), which aligns with literature indicating that it is generally activating rather than sedating at therapeutic doses [41].

Gastrointestinal tolerability received mixed responses. While many respondents rated duloxetine as better tolerated than other antidepressants, some perceived more GI side effects. This perception is supported by the literature, which shows duloxetine has a lower incidence of GI issues than venlafaxine but more than placebo [42].

Regarding use in older adults (aged 50–75), more than half of the respondents considered duloxetine safer than other SNRIs, with the remainder rating it equally safe. No respondents considered it less safe, a finding supported by evidence showing duloxetine’s acceptable safety profile in older populations [18,43,44].

Perceptions of adherence were also positive: 69% of psychiatrists believed that adherence to duloxetine was better than with other antidepressants, while 31% found it comparable. Studies show higher adherence and persistence with duloxetine in MDD and chronic pain compared to escitalopram and venlafaxine [45], with similar trends in GAD [46]. A 2021 prospective multicenter study demonstrated significant positive effects on illness severity and quality of life when patients were switched to duloxetine from other antidepressants [47]. This suggests that improved tolerability and efficacy contribute to better treatment persistence.

Regarding combination therapy, 80% of respondents viewed combining duloxetine with other antidepressants positively, and only 4% expressed a negative view. While combination strategies must be carefully monitored for safety, existing research suggests that such regimens can be as effective and safe as monotherapy when appropriately managed [14,48]. These data highlight duloxetine’s versatility in combined treatment regimens, which is especially relevant for patients with treatment-resistant symptoms.

Satisfaction rates were high: 99% of psychiatrists reported being “satisfied” or “very satisfied”, with only 1% neutral and no negative responses. This likely reflects duloxetine’s perceived efficacy, safety, and tolerability in real-world use.

Finally, the fact that 100% of respondents would recommend duloxetine—74% “definitely” and 26% “probably”—further underscores its strong reputation in psychiatric practice. This level of support highlights its versatility and reliability in treating a range of psychiatric and somatic conditions.

Collectively, the survey results demonstrate remarkable alignment between clinical practice and scientific evidence. Recent literature continues to support duloxetine’s multi-modal benefits, particularly in patients with comorbid pain and mood symptoms [49]. The psychiatrists’ positive experiences reflect the medication’s unique dual serotonin-norepinephrine mechanism, which provides advantages in treating both emotional and somatic symptoms of depression [50].

Contemporary research directions include exploring duloxetine’s role in cognitive enhancement [51], its potential in treatment-resistant depression [52], and its cardiovascular safety profile in elderly populations [53]. The integration of real-world evidence with controlled trial data continues to support the clinical observations reflected in this psychiatrist survey.

The convergence of clinical experience and scientific evidence suggests that duloxetine maintains its position as a versatile, effective, and well-tolerated treatment option across multiple psychiatric and pain conditions, validating the positive perspectives expressed by the surveyed psychiatrists. It is important to reiterate that the findings of this study reflect psychiatrists’ subjective perceptions rather than measured prescribing behaviors or patient-level clinical outcomes. As such, while these perceptions may align with trends in the literature, they should be interpreted within the context of self-reported, descriptive data.

### Limitations

This study has several limitations that should be acknowledged. First, the absence of clinical efficacy data—such as standardized symptom scales or remission rates—precludes direct comparison between perceived and objective outcomes. While our primary aim was to assess clinician perceptions, future studies should integrate patient-level clinical metrics to provide a more comprehensive evaluation of effectiveness.

Second, the sample was limited to 80 psychiatrists, which may not fully represent the diversity of clinical practice across different regions or healthcare systems. As such, generalizability is limited, and broader, more geographically diverse samples should be considered in future research.

Third, this study did not include patient-reported outcomes or medication adherence data directly from patients. These aspects are essential in real-world evaluations and should be prioritized in future studies.

Fourth, the study did not include a comparator analysis against other antidepressants or pain agents. While the focus was on duloxetine, the inclusion of comparator data would allow better contextualization of its efficacy, safety, and tolerability.

Fifth, the uniformly positive evaluations reported across most items are noteworthy and suggest strong professional confidence in duloxetine. However, such consistency also raises the possibility of response bias or social desirability effects, which may have influenced psychiatrists to report more favorable views. While the survey was anonymous and voluntary, the potential for such biases remains an important consideration and underscores the need for complementary research using patient-reported outcomes and objective clinical data.

Sixth, the analysis relied exclusively on descriptive statistics, as no subgroup or demographic variables (e.g., years of clinical experience, practice setting) were collected that would allow for inferential comparisons. This was consistent with the exploratory and perception-based design of the study. Future research could benefit from incorporating stratified data to allow for subgroup analysis or hypothesis testing.

## 5. Conclusions

The results of this study reflect a highly positive perception of duloxetine among the psychiatrists surveyed, supporting its integration into clinical practice as an effective and well-tolerated option for the treatment of MDD, particularly in patients with chronic pain. These perceptions align with existing scientific literature that supports the utility of duloxetine in MDD and other conditions such as neuropathic pain and GAD. Furthermore, its perceived safety and tolerability profile, along with reported favorable adherence rates, contribute to the high level of satisfaction among healthcare professionals. The absence of negative responses on key aspects—such as adherence and willingness to recommend the drug to colleagues—further reinforces the reported confidence in duloxetine and its relevance as perceived by psychiatrists in the management of psychiatric disorders. However, it is important to emphasize that these findings are based on self-reported attitudes and do not constitute direct clinical efficacy data.

Future research should aim to integrate objective clinical outcomes, including standardized symptom scales, remission rates, and patient-reported outcome measures, to evaluate whether the perceptions observed in this study are supported by patient-level evidence. Longitudinal studies incorporating patient-reported outcomes and adherence data will provide a more comprehensive understanding of duloxetine’s real-world effectiveness. Expanding the sample size and including a more diverse population of healthcare professionals across various regions and healthcare systems would improve the generalizability of findings. Comparative studies involving duloxetine and other antidepressants or neuropathic pain agents—especially in monotherapy versus combination therapy contexts—are needed to better contextualize its relative benefits and tolerability. Additionally, exploring the pharmacoeconomic implications of duloxetine use in daily practice may offer valuable insights into its cost-effectiveness, aiding clinical and policy-level decision-making. Finally, further investigation into the mechanisms behind variable responses in different patient subpopulations, such as those with GAD or somatization, could help personalize treatment strategies and optimize outcomes.

## Data Availability

Data are available upon request to the corresponding author, but access is restricted because participants consented only to analyses conducted by the primary research team.

## Supplementary Materials

The following supporting information can be downloaded at https://www.mdpi.com/article/10.3390/brainsci15070757/s1, Questionnaire.
