# Supplementary material for: Delving into the Perception, Use, and Context of Duloxetine in Clinical Practice: An Analysis Based on the Experience of Healthcare Professionals
Source: Brain Sci. 2025 Jul 17;15(7):757. doi: 10.3390/brainsci15070757 (PMC12293169; doi:10.3390/brainsci15070757)
Supplement: Supplementary file 1 [file brainsci-15-00757-s001.zip › brainsci-3731541-supplementary.pdf]

N= 80
20 Questions

1) How familiar are you with the use of duloxetine in your clinical practice?
a) Very familiar
b) Quite familiar
c) Somewhat familiar
d) Slightly familiar
e) Not at all familiar
Responses A1: (80%, 20%, 0%, 0%, 0%)

2) How often do you recommend duloxetine for patients with comorbid major depressive disorder (MDD) and chronic pain?
a) Always
b) Frequently
c) Occasionally
d) Rarely
e) Never
Responses B2: (26%, 73%, 1%, 0%, 0%)

3) How often do you prescribe duloxetine for major depressive disorder (MDD)?
a) Frequently
b) Occasionally
c) Rarely
d) Never
Responses C3: (90%, 10%, 0%, 0%)

4) How would you compare adherence to duloxetine treatment versus other antidepressants?
a) Much better
b) Better
c) The same
d) Worse
e) Much worse
Responses D4: (13%, 56%, 31%, 0%, 0%)

5) Compared to other antidepressants, how would you rate the effectiveness of duloxetine in treating neuropathic pain?
a) Much more effective
b) More effective
c) Equally effective
d) Less effective
e) Much less effective
Responses E5: (30%, 63%, 6%, 1%, 0%)

6) Have you ever had to discontinue duloxetine treatment due to side effects?
a) Frequently
b) Occasionally
c) Rarely
d) Never
Responses F6: (0%, 51%, 45%, 4%)

7) Which of the following antidepressants causes the least weight gain?

a) Duloxetine
b) Amitriptyline
c) Paroxetine
d) Mirtazapine
Responses G7: (97.5%, 0%, 2.5%, 0%)

8) What is your perception of the risk of serotonin syndrome with duloxetine compared to other SNRIs?
a) Much higher
b) Higher
c) Same
d) Lower
e) Much lower
Responses H8: (0%, 0%, 51%, 41%, 8%)

9) Which of the following antidepressants produces fewer sexual side effects?
a) Sertraline
b) Venlafaxine
c) Duloxetine
d) Escitalopram
Responses I9: (6.25%, 2.5%, 82.5%, 8.75%)

10) What is your opinion on the use of duloxetine in combination with other antidepressants?
a) Very favorable
b) Favorable
c) Neutral
d) Unfavorable
e) Very unfavorable
Responses J10: (10%, 70%, 16%, 4%, 0%)

11) Which of the following antidepressants causes the least sedation?
a) Sertraline
b) Duloxetine
c) Paroxetine
d) Amitriptyline
Responses K11: (31%, 69%, 0%, 0%)

12) How would you rate the efficacy profile of duloxetine in treating generalized anxiety disorder (GAD) compared to other SNRIs?
a) Much more appropriate
b) More appropriate
c) Equally appropriate
d) Less appropriate
e) Much less appropriate
Responses L12: (6.25%, 42.5%, 48.75%, 2.5%, 0%)

13) In your experience, how effective is duloxetine in treating somatization?
a) Very effective
b) Effective
c) Moderately effective
d) Slightly effective
e) Not effective
Responses M13: (20%, 50%, 27.5%, 2.5%, 0%)

14) What is your overall level of satisfaction with the use of duloxetine in your practice?
a) Very satisfied
b) Satisfied
c) Neutral
d) Dissatisfied
e) Very dissatisfied
Responses N14: (49%, 50%, 1%, 0%, 0%)

15) How would you compare the gastrointestinal side effects of duloxetine with those of other antidepressants?
a) Much more severe
b) More severe
c) Equally severe
d) Less severe
e) Much less severe
Responses O15: (0%, 8%, 21%, 66%, 5%)

16) Would you recommend duloxetine to other psychiatrists as a viable treatment option for their patients?
a) Yes, definitely
b) Yes, probably
c) Not sure
d) Probably not
e) Definitely not
Responses P16: (74%, 26%, 0%, 0%, 0%)

17) Have you had patients report an improvement in quality of life with duloxetine?
a) Yes, all
b) Yes, many
c) Yes, some
d) Few
e) None
Responses Q17: (1%, 83%, 16%, 0%, 0%)

18) How would you rate the efficacy of duloxetine for diabetic peripheral neuropathic pain compared to other SNRIs?
a) Much more effective
b) More effective
c) Equally effective
d) Less effective
e) Much less effective
Responses R18: (33%, 61%, 6%, 0%, 0%)

19) What is your opinion of the overall side effect profile of duloxetine?
a) Completely acceptable
b) Mostly acceptable
c) Moderately acceptable
d) Slightly acceptable
Responses S19: (35%, 59%, 6%, 0%)

20) How would you rate the safety profile of duloxetine in older patients (aged 50–75) compared to other SNRIs?
a) Much safer
b) Safer

c) Equally safe
d) Less safe
e) Much less safe
Responses T20: (15%, 56%, 29%, 0%, 0%)
